# Spiritualism and Hypnotism

**Published:** 1885-12

**Authors:** G. C. Paoli

**Affiliations:** Chicago, Emeritus-Professor Woman’s Medical College; 502 Webster Avenue


### OBSERVATIONS ON SPIRITUALISM AND HYPNOTISM.

*By* G. C. PAOLI, M. D., *Chicago, Emeritus-Professor Woman's Medical College.*

[Read before the Chicago Medical Society.]

We live in a progressive period, in a time remarkable for new scientific discoveries, new mechanical inventions, new philosophical theories. Many natural phenomena which formerly were considered mysterious, can now be explained on scientific principles. We are no longer satisfied with effects, we must examine into causes, and the deeper we investigate, the less mysterious do our surroundings appear, and we discover that our planet, and all that on it, moves, is governed by immutable natural law, simple, easily understood, for, as a Swedish poet has beautifully said, " All things high and holy, both in science and poetry, are so simple that a child can understand them." In former times thunder and lightning were supposed to be caused by the meeting of a warm and cold cloud, but we have since learned that this phenomena is caused by electricity. So spirit-rappings were believed to be the medium of communication with the spirit world, but calm, thorough investigations have convinced us that such communications only occur at the meetings of a rogue and a simpleton. Superstition has always held a strong hold on the mind, and even the powerful light of science has not yet dispelled it away from civilized lands, although its forms change with changing times and circumstances. In ancient times the priests determined the future by the flights of birds, the interpretation of dreams, etc., and oracles and astrology fostered superstition, and the priesthood never lost an opportunity to profit by the eager desire of mankind to penetrate the veil that concealed the future. This kind of superstition disappeared before the light of progress, but another kind of prophets arose, who foretold the future by cards, lines in the hand, etc. But these were not satisfactory to the thinking mind; little by little this art fell into disrepute, though there are still at this day too many who believe in like arts of the fortune-tellers. In our days a new superstition has arisen, called Spiritualism, which has now so many believers that they too form a new religious sect, whose members may sit around a table, and call up good and evil spirits at will. When spirit-rappings were first spoken of in this country there was a diversity of opinion as to how it was done, some being unwilling to believe that the raps were produced by spirits, while others believed them to be a sort of magnetic or electric operation, intended to deceive people and rob them of their money. The first who practiced these knockings, called themselves spiritual mediums, were the Fox girls of Rochester, N. Y., who made a very lucrative business of it. Some thought the raps were produced by the friction of the fingers, other that they were the results of new chemical processes. But on closer examination none of these suppositions were satisfactory, but what appeared to be a mystery proved to be in reality but as base a deception as ever has been perpetrated in a civilized land, a superstition which is a disgrace to the 19th century; resembling the Salem witchcraft and quite as injurious in its effects upon the community. Let us first examine into the causes which actuate people to impose upon the confidence of their fellow men. Some are actuated by a love of gain, others of notoriety. A third class, though undoubtedly honest in their belief, are of nervous temperament, vivid imagination, exceedingly superstitious, and easily carried away by every new issue, and particularly ready to be influenced by every thing that is beyond their comprehension. Having investigated the subject thoroughly, I will, as concisely as possible, give the result of my investigations. At an exhibition given by one of the powerful mediums, who claims to be able to move tables and other furniture by laying on of hands, I discovered that the wonder was wrought by muscular power alone. The medium in this case was a strong, muscular young woman, who, to the astonishment of the company assembled to witness her performances, pushed not only chairs before her, but the persons also who laid their hands upon them. Watching them closely, I observed that they did not at all oppose her strength, but merely kept their hands on the object while she pushed them along without their noticing it I determined to try what effect it would have to oppose my strength directly to hers. The consequence was that she could neither move the table nor the persons whose hands were on it, which created no little merriment amongst the spectators, and surprise and chagrin to the medium. She exerted herself to push the table along till great drops of perspiration rolled down her face, and after trying in vain, she finally gave up in despair.

Others, calling themselves writing-mediums, claim to be able to receive and write down communications from departed spirits. After calling up the spirit from whom the communication is desired, the hands of the medium are placed on a little table, which is tipped or rapped on, in response to the question, Is their a spirit present? and the medium is prepared with writing materials to take down the communication, and becomes very nervous, the hands tremble, unsuccessful attempts are made to write, till finally their comes a vague, unsatisfactory answer, often very far from the desired reply. For instance, if I ask for the name of one of my deceased parents, the medium writes your mother.

If I persist in asking for the name, the medium writes below that, I am the spirit of your mother, or some equally vague reply, which is very unsatisfactory to me, but perfectly so to those who are inclined to the belief, and have no doubts of the medium's truthfulness. If I still insist on a more satisfactory answer, the medium will write, "You are faithless and unbelieving," or, "Mind your own business," or something similar, whereupon the medium or some believer will say, "There is an evil spirit present which is disturbing the communication from the departed friend." In other words, when the good spirit is fairly cornered, the discomfiture is laid to the interference of the evil one, thus leading the inquisitive mind to another subject till he is prepared for other communications, which, from the medium's knowledge of his history and relations may be correct; then we are informed that the evil spirit is no longer present. When a correct answer is given the believers seize and enlarge upon it, but never mention when mistakes are made. I have witnessed many of these manifestations and they all remind me of the ceremonies of the Egyptians, who worshiped various animals, particularly the ox. When they desired information concerning the future they consulted this animal and accepted the movements of his head or tail as decisive answers to their questions. In ancient times the priests were physicians, the temples were the dispensaries; the legends of the saints, the history of witchcraft, charms, amulets and relics, all taught that all kinds of diseases were cured by their magic means. But although spiritualism has arisen in our times, the tricks of those who have imposed upon the public have been exposed by so many of the most renowned mediums, such as the Davenport Brothers and others, that its believers are now steadily decreasing. Hypnotism, which is derived from a Greek word signifying sleep, and is usually known as mesmerism. One century ago Dr. Mesmer imposed on the public in France with a power which he called animal magnetism, by which he pretended to cure diseases, but after thorough investigation by several scientific men, it was pronounced a fallacy and an imposition. After residing in Paris sometime he gradually lost the esteem and confidence of the public and returned to Switzerland to enjoy the large sum of money he had accumulated in France, but never after his return to his native land did he pretend to cure diseases with animal magnetism or so called mesmerism. Later, Dr. Beard, of England, who was by no means a pretender, but rather an enquirer after truth, experimented with the magnet in o-der to see whether it had the power as many supposed. And instead of using the magnet, he often took a piece of wood instead, and the process whereby he succeeded in producing sleep was simply to cause the person under control to fix the eyes steadily on a certain object; this he called Hypnotism, or Sleep.

During my residence in Stockholm, the capital of Sweden, I had an excellent opportunity to become thoroughly informed on all the phenomena of hypnotism. A Swedish general, who had served in the Turkish military service and had been honored with the title of Pasha, returned to his native country financially destitute. Trying to do something for his support, he applied himself to making experiments in Hypnotism. I was invited not only to see but to observe in detail the subjects he practiced on. The individuals he influenced were generally extremely nervous young subjects, and in many cases he failed. When he addressed them he generally did so in a commanding or authoritative tone. When any presented themselves who had wills of their own, he never succeeded. During the operation he always requested all to fix their gaze upon his eyes. I was satisfied from observation that some of the faculties of the mind were suspended for a few minutes, and the individuals he experimented on were weak-minded and credulous. A healthy and sound mind is characterized by the proper balance of all mental faculties in the same manner that a healthy body is dependent on the proper action of all the nerves. There are mental illusions and sensorial illusions caused by predominant ideas and corrected by proper reasoning. As an instance of a false impression I will relate a case taken from an English medical journal, namely: A lady in England who one summer day enjoyed a sleep in her garden, dreamed that a grass snake had entered her bowels, after which she lived in the hallucination that she felt a strange motion, which she claimed was produced by the snake. She went to several physicians to obtain medical aid which would destroy the animal, but all in vain. In the meantime a physician declared that her diagnosis was correct, and he produced a small, innocent snake, which he had concealed, and during his examination he pretended to extract the animal, and by showing it to her she was perfectly cured, and complained only of the other doctors' ignorance. To the reflecting mind it seems that these abnormal ideas are like all religions with their gods, their demigods, their prophets and their saints, which were created by the credulous fancy of men who had not attained to the full development and possession of their intellectual faculties. Still, with all the ignorance and superstition which surrounds us, the greatest science of the day, namely, evolution, has done more for the intellectual development of the world than anything else. Such illustrious men as Huxley, Darwin, Haeckel and Lyell have not labored in vain. Their first attempts were ridiculed and despised by the orthodoxy, but their investigations stand to day as a mathematical fact, and will in the end unite the people of the world by grander, nobler principles, namely, a general scientific education, self-sacrifices and devotion to humanity.

502 Webster Avenue.

